# H5N1 2.3.4.4b: a review of mammalian adaptations and risk of pandemic emergence

**DOI:** 10.1099/jgv.0.002109

**Published:** 2025-06-04

**Authors:** Fernando Capelastegui, Daniel H. Goldhill

**Affiliations:** 1 Department of Pathobiology and Population Sciences, Royal Veterinary College, Hatfield, UK

**Keywords:** adaptive mutations, avian influenza, influenza, mammalian adaptation, pandemics, spillover

## Abstract

Avian influenza viruses can cause severe disease when they spill over into mammalian and human hosts. H5N1 clade 2.3.4.4b has spread globally since 2021, decimating avian species, and has spilled over into mammalian species, causing sporadic infections and fatal outbreaks in sea lions, cats, mink and dairy cattle. Increased human cases of H5N1 are fuelling concern that H5N1 could soon adapt to become a new pandemic virus. Adaptive mutations have emerged following spillover, which support H5N1 outbreaks in mammalian populations and include changes to the PB2 such as E627K, D701N, M631L and T271A. Further changes to haemagglutinin, altering binding preference to human-like *α*2,6 sialic acid receptors have yet to be seen. Here, we review the adaptations that have emerged in mammals throughout the 2.3.4.4b outbreak and the molecular mechanisms behind these mutations to assess the pandemic risk of this virus.

## Introduction

Avian influenza viruses (AIVs) are a major cause of disease and mortality in both wild birds and livestock. Although AIVs primarily affect avian species, spillover events into mammals and humans regularly occur, often resulting in death [1]. Adaptation of AIVs to mammalian hosts poses a significant public health threat and could lead to the emergence of a novel strain capable of causing a new pandemic [1].

AIVs pose an increased risk to global health given the widespread circulation of highly pathogenic avian influenza H5N1 clade 2.3.4.4b. This virus has spread worldwide, infecting an unprecedented number of avian and mammalian species. The consistent detections in mammals, including mass mortality events, mark a step change in the risk this virus poses both from an animal health and public health perspective [1,2]. The outbreak in dairy cattle in the USA and linked human cases have especially piqued concerns as the virus has adapted to a new mammal host and spread effectively through herds across the country.

This review will examine the mammalian incursions and adaptations seen in the H5 2.3.4.4b outbreak, focusing on the molecular biology underlying AIV infection in mammals. By examining the mechanisms of adaptation to mammals, we evaluate the pandemic potential of H5 viruses in birds and mammals.

### The origin and history of the H5N1 2.3.4.4b outbreak

The virus causing the current H5 outbreak originated in China where the Highly Pathogenic Avian Influenza H5N1 A/goose/Guangdong/1/1996 (GsGd) lineage was first identified in 1996. This lineage spread globally and diversified, enabling it to infect both wild and domestic bird populations episodically across the globe [3,5]. Clade 2 has been the most successful of the GsGd lineage, giving rise to several subtypes that have circulated in Africa, Asia, Europe and North America. Clade 2.3.4.4, which can be further split into eight subclades (a–h) [5], has been dominant since 2008 and gave rise to the prevailing subtypes H5N2, H5N5 and H5N8 which circulated prior to 2014, causing significant losses to predominantly domestic bird populations [6]. In more recent years, between 2014 and 2021, H5N6 and H5N8 reassortants caused sporadic outbreaks, again largely in captive birds.

AIVs can be split into high and low pathogenicity avian influenza (HPAI and LPAI, respectively), based on mortality rates in chickens, though broadly HPAI are more lethal than LPAI viruses. The current H5 outbreak is the most widespread HPAI panzootic in wild birds. A key turning point occurred in 2020/21 when the dominant H5N8 2.3.4.4b was reassorted with LPAI Eurasian viruses, resulting in an H5N1 subtype with a unique gene constellation. First detected in the Netherlands, this H5N1 virus caused a severe outbreak in birds across Europe during that period. Unlike the preceding H5N8 outbreaks of earlier years, this H5N1 caused mass mortality events in previously unaffected wild bird species as well as many domestic flocks. During this outbreak, H5N1 dominated over other H5Nx subtypes [4] and emerged in new species and territories and, unusually for an HPAI, persisted in avian populations year-round. There has since been widespread mortality in bird populations around the globe including sea bird colonies, waterfowl and peridomestic birds such as pigeons and crows as well as millions of poultry which have been culled.

### Mammals

**Fig. 1.**
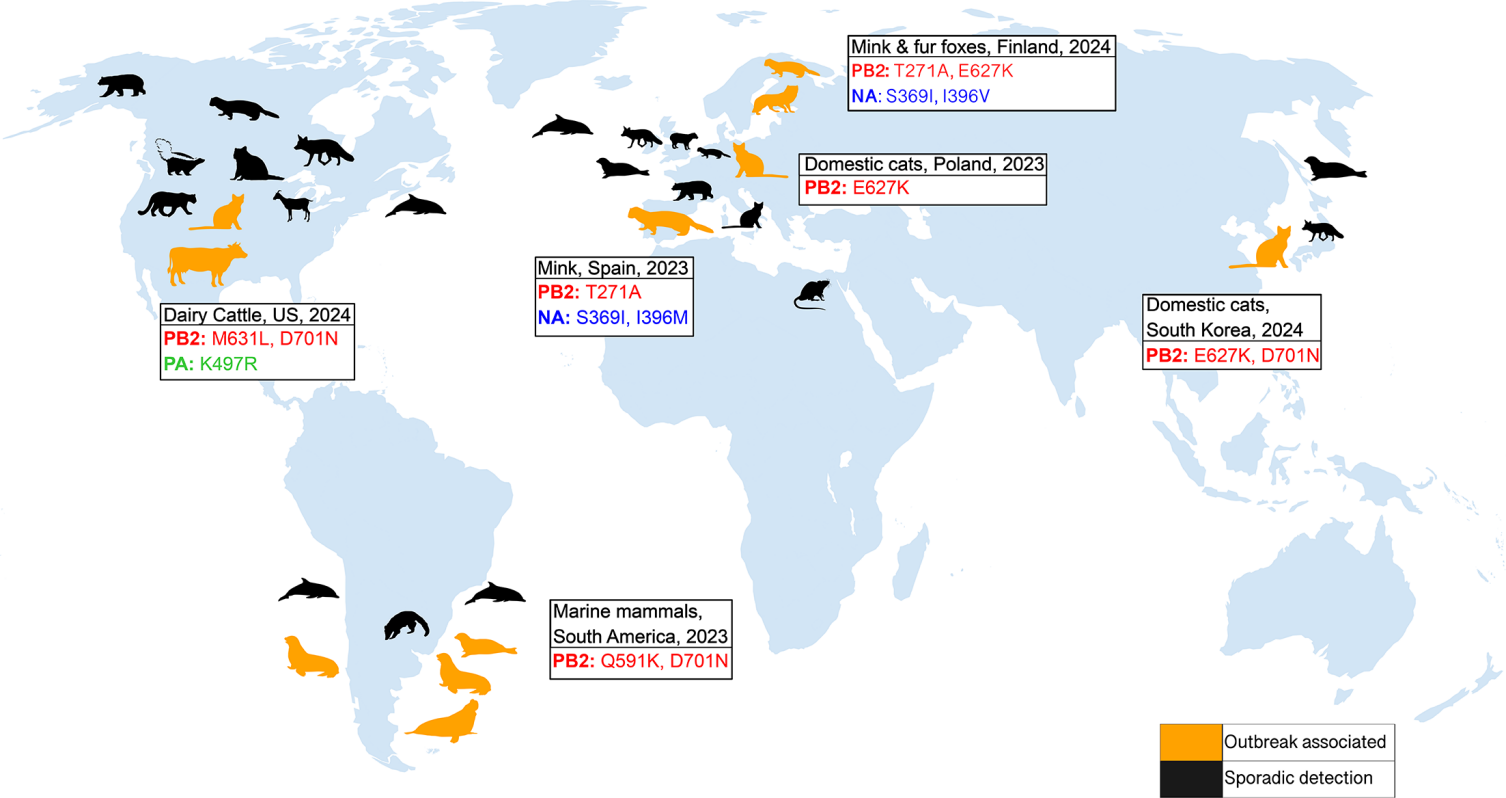
Global detections of A(H5N1) clade 2.3.4.4b in mammalian species, split by sporadic detections (black) and detections which are associated with an outbreak (orange) alongside key mammalian adaptive mutations. Note: ‘outbreak’ does not imply mammal-to-mammal transmission but common epidemiological and environmental exposures. See Table S1 (available in the online Supplementary Material) for species identification key. Silhouettes from PhyloPic (www.phylopic.org).

A key characteristic of the H5N1 2.3.4.4b panzootic is the wide range of infected mammalian hosts (Fig. 1). The signs of disease vary between mammals, with symptoms including respiratory, neurological and systemic illness [1]. Since the start of the outbreak, sporadic detections in wild mammals including foxes, otters and other carnivorous species such as polecats [7,10] have been reported across Europe. These detections are mainly through sampling dead or sick wild animals, and infections have largely been attributed to the scavenging of diseased wild birds, though some environmental transmission (e.g. from infected water sources) is possible [11,12]. Throughout the outbreak, semi-domestic and captive animals have also been infected, for example, a bear in a French zoo, as well as bush dogs in the UK [13,14]. European marine mammals including dolphins and seals were also found to be infected, highlighting the wide diversity of species and habitats affected [15,16].

Larger scale mass infection and mortality events have also been reported where mammal-to-mammal transmission likely occurred unlike in the sporadic detections listed above. In 2022, an outbreak was detected in Spain within farmed mink [17], with similar outbreaks occurring across Finland in 2023 [18] following a spillover event from wild birds (both genotype EA-2022-BB). Cats were also affected in Poland [19] and South Korea [20] in 2023, with the source of infection thought to be contaminated raw food derived from poultry. One of the most devastating developments was the mass die-off of marine mammals in South America where hundreds of thousands of seals, sea lions and elephant seals perished [21,25] (genotype B3.2). Recently, in early 2024 in TX, USA, H5N1 genotype B3.13 was identified in dairy cattle, which were previously not thought to be carriers of AIVs [26,27]. B3.13 is a reassortant of European HPAI H5N1 and North American LPAI [1]. The virus is thought to be spread between cows during milking, with infections mainly seen in the udder. Influenza spreads between farms due to the movement of cattle, people and contaminated equipment and has infected herds of dairy cattle across the country continuing into 2025. Unlike in many other mammals, H5N1 currently presents as a mild illness in cows characterized by a drop in milk production and mastitis. Recent reports from the USA have seen further independent incursions into cattle of genotype D1.1, a separate reassortant from B3.13 of HPAI and LPAI [28]. These infections were originally identified through testing of bulk milk, suggesting that the transmission between wild birds and cows may happen more frequently than previously thought [29].

### Humans

The WHO reports a total of 964 human cases of H5N1 with a case fatality rate (CFR) of 48.3% [30]. The CFR for H5N1 2.3.4.4b viruses is comparatively low; however, as of February 2025, there have been 91 human cases, with 2 associated deaths. The predominant symptoms of B3.13 cattle outbreak infections have been conjunctivitis and mild respiratory illness. The D1.1 genotype, whilst also predominantly mild, has caused two severe cases in humans: one in British Columbia, Canada, and one fatal case in LA, USA [31,32]. Additionally, a severe case in Chile in 2023 (genotype B3.2) reaffirms that this virus can cause serious outcomes [30]. Human cases in Cambodia of a genetically distinct H5N1 clade 2.3.2.1 .c also appear to be more severe, though the determinants of severity between clades and subtypes remain unclear and challenging to ascertain from sporadic infections. Underreporting and surveillance differences may lead us to overestimate severity and underestimate true case rates of H5N1. A recent study of dairy farm workers in the USA found that up to 7% of individuals may have antibodies to H5N1 [33], suggesting unchecked occupational exposure and undetected infections. Similarly, proactive surveillance of asymptomatic exposed persons led to early detection of cases in Spain and the UK [34,36], though the extent to which these infections were true infections and not contaminations is unclear. The risk of exposure to H5N1 has increased however for the public following the detection of H5N1 in raw dairy products in the USA. Although no definitive infections via this route have been reported, there is anecdotal evidence cats have been infected through raw milk consumption [37].

**Fig. 2.**
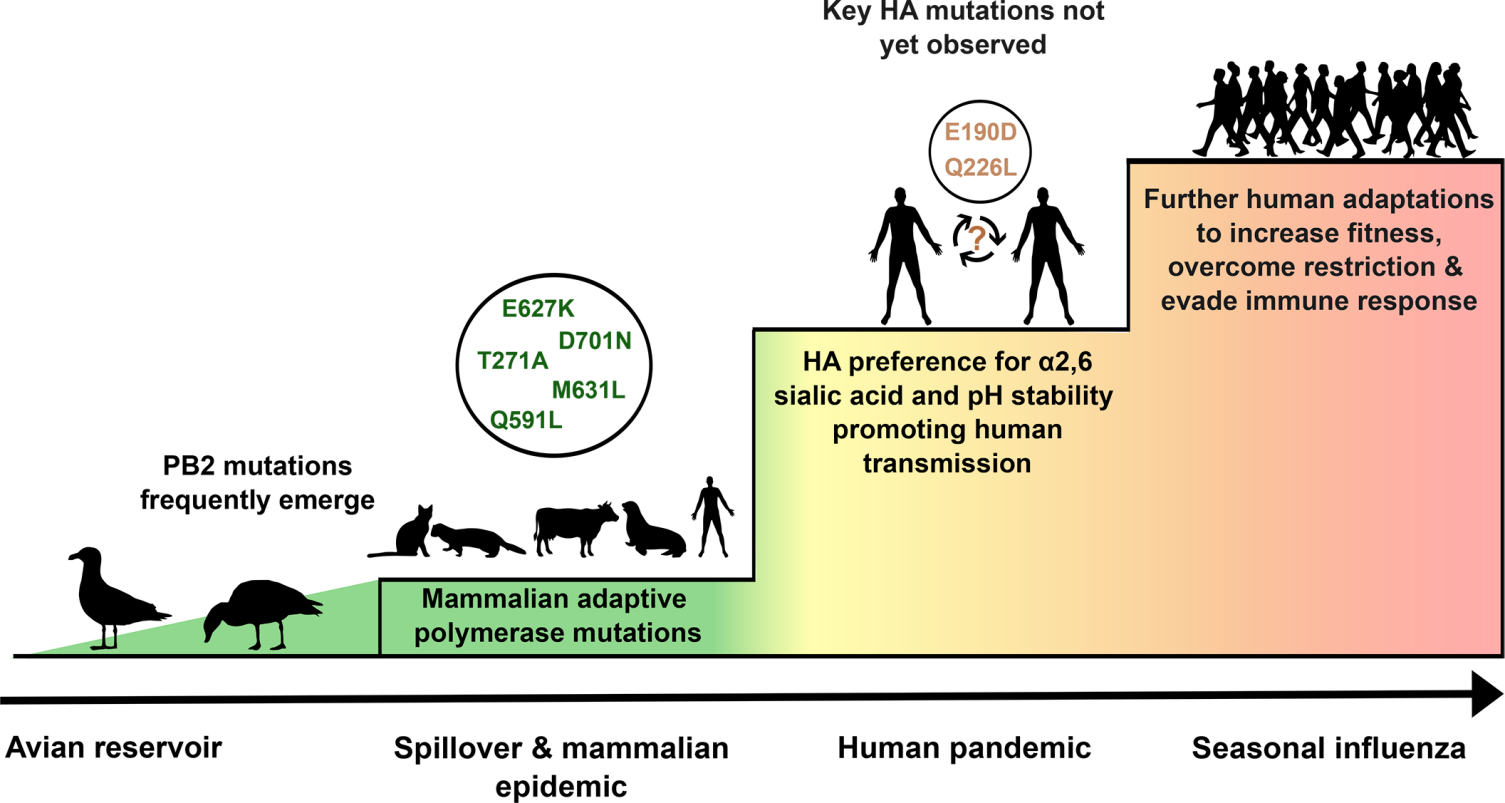
Pandemic staircase adapted from Long *et al*. 2019 [43] showing the transition of AIV to the human seasonal virus. Key steps for mammalian and human adaptation are polymerase changes, HA mutations which confer *α*2,6 sialic acid preference and finally fine-tuning of viral fitness. Species silhouettes from PhyloPic (www.phylopic.org). A comprehensive review by Suttie *et al*. [92] of known mammalian adaptive mutations and their functions, including those discussed in this review, can be found here: https://pmc.ncbi.nlm.nih.gov/articles/PMC6831541/#Sec13.

To date, there has been no evidence of sustained human-to-human transmission of H5N1 2.3.4.4b. However, mammal-to-human transmission and continuing detections in mammals are fuelling serious concern that as this virus continues to adapt, it could become a virus with pandemic potential. The ‘pandemic staircase’ proposed by Long *et al*. sets out how an avian virus becomes a pandemic strain (adapted in Fig. 2), starting with polymerase adaptations, changes to the haemagglutinin (HA) and finally fine-tuning to human hosts. Here, we set out the molecular mechanisms and biology of mammalian adaptations along this framework to assess the pandemic risk of this virus.

## Mammalian adaptations of H5N1 2.3.4.4B

### The viral polymerase and essential host factors

The influenza polymerase uses host factors inside the cell [38,39] with proteins from the ANP32 family essential for polymerase activity [40,44]. It is thought that ANP32 proteins bridge asymmetric polymerase dimers, mediating and stabilizing conformational changes needed for cRNA→vRNA (complementary RNA→viral RNA) during genome replication [45,46]. AIVs rely on avian ANP32A, which, due to an exon duplication between the leucine-rich repeat motif and low complexity acidic region (LCAR), has an extra 33 aa compared with mammalian ANP32A and ANP32B [47]. Crucially, ANP32A presents a major host-species barrier, as avian-adapted polymerases cannot efficiently use the shorter mammalian ANP32A/B. In avian ANP32A, the LCAR contains mixed charges; however, in mammalian homologues, the lack of these 33 aa results in an entirely acidic region [45]. Mammalian ANP32 proteins therefore require a positively charged groove to bind and stabilize the polymerase dimer [48].

**Fig. 3.**
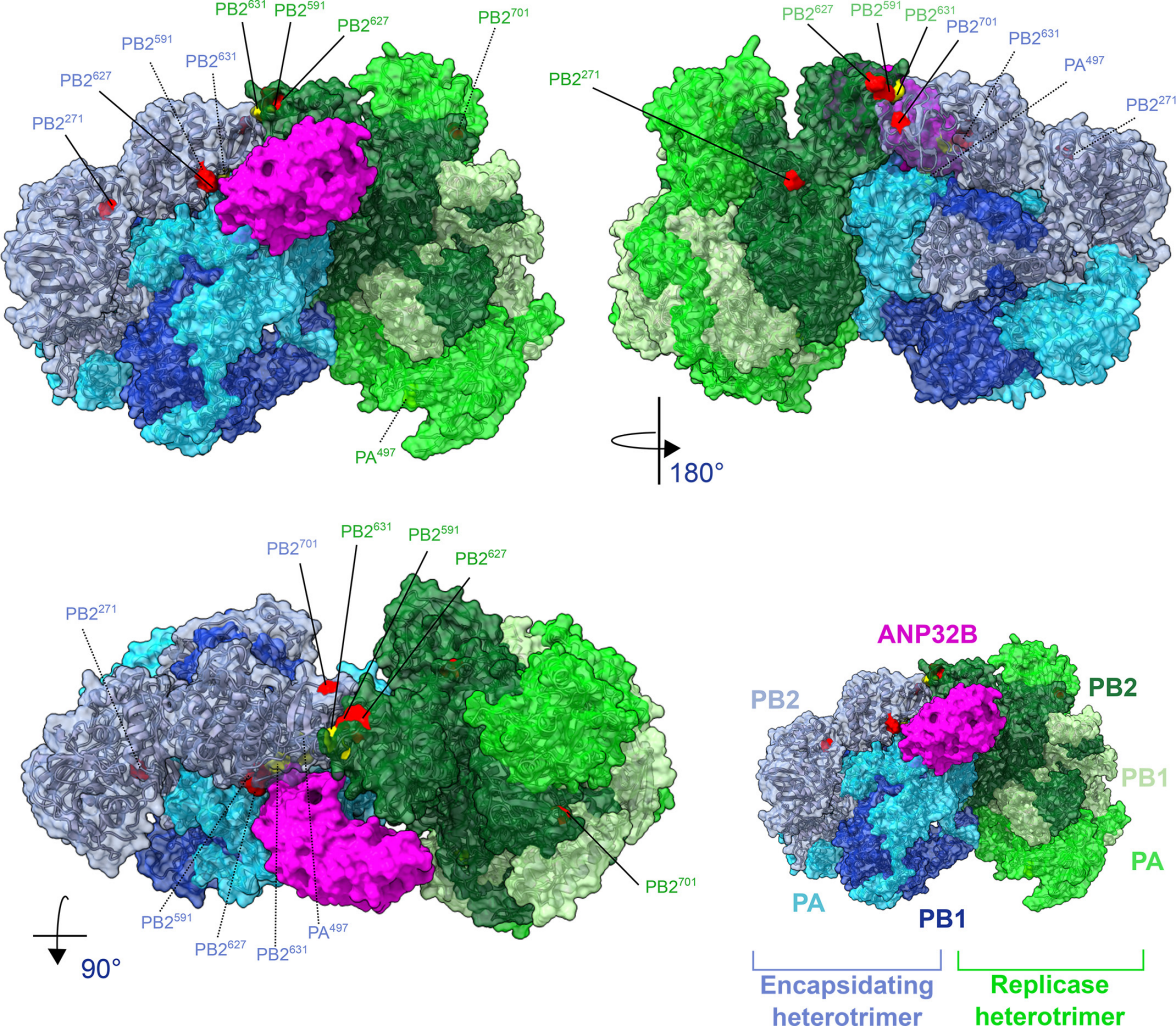
H5N1 influenza polymerase dimer complex bound to human ANP32B. Key mammalian adaptive mutations are highlighted in red, and bovine-specific emergent mutations are highlighted in yellow. PDB: 8R1J [45]. Protein structure graphic created using ChimeraX version 1.9 [149].

The most common mammalian PB2 mutation is E627K. This residue change from acidic to basic complements the acidic nature of the binding region in human ANP32 LCAR described above, allowing it to bind effectively [48] (see Fig. 3). Other mutations such as PB2 Q591R and D701N are also commonly observed and adapt avian viruses to use mammalian ANP32 proteins through similar biochemical changes to E627K. Furthermore, PB2 E627K favours ANP32B proteins, whereas Q591R and D701N are not ANP32 homologue specific, explaining why different mutations may arise in the context of specific adaptation to host-specific differences in ANP32 proteins [47]. In addition to interacting with ANP32, D701N has also been associated with the increased import of vRNPs (viral ribonucleoproteins) into the nucleus by exposing the nuclear localization signal in PB2, which promotes importin-*α* interactions [49]. T271A is another key mutation in PB2 and boosts polymerase activity, although not as effectively as E627K [47]. Alongside Q591R, T271A can further compensate for the lack of E627K [50]. It is hypothesized that T271A stabilizes the asymmetric polymerase dimer in an encapsidating conformation, though the full adaptive mechanism behind T271A remains unknown [45,47, 51, 52].

E627K, T271A and D701N have not been widely seen in the B3.13 US cattle outbreak, suggesting other mutation profiles are enabling the recruitment of bovine ANP32 proteins [12,27, 53, 54]. The key adaptive mutation found in cows was PB2 M631L, which is structurally close to PB2 627 and 591 [55,56]. Also emerging was PA K497R, which is located close to this interface [56] (see Fig. 3). Recent experimental data in bovine and human cells shows that PB2 M631L is the main actor in restoring polymerase function with potentially a small boost from PA K497R [56]. Further mutations may also help to increase polymerase activity such as PA mutations I13V and E613K seen on a minor clade of the cattle outbreak and D740N, which has arisen multiple times independently in the outbreak [56].

Experimentally, *in vitro*, E627K is still more effective than M631L and PA K497R [56]. Thus, it remains unclear why this mutation was fixed instead of E627K, especially considering that the first human B3.13 infection had E627K. Interestingly, D1.1 cattle viruses have not contained PB2 E627K, with one containing D701N, suggesting that E627K may not be strongly selected for in cows [57]. Which exact mutations emerge may be a stochastic process and driven by differences in bovine ANP32s. Indeed, when tested with different ANP32 homologues, M631L favoured bovine ANP32A over ANP32B, whereas E627K has been shown to prefer ANP32B, suggesting that selection to use bovine ANP32A is driving the mutations seen in the cattle outbreak. M631L complements not only bovine ANP32s but also swine and human homologues, suggesting that it is an adaptation beneficial across mammalian species.

Outbreaks seen in mammals can take off following *de novo* adaptation of the polymerase to ANP32 following spillover from a bird, with key mutations PB2 E627K, Q591R and D701N arising frequently across many species both wild and captive (Fig. 1). In marine mammal sequences from South America, we see PB2 D701N and Q591K in combination with other mutations [21,22, 25, 58]. In contrast, an outbreak in captive mink in Spain was characterized by T271A. Similar but separate mink outbreaks in Finland saw T271A and E627K emerge, albeit in different farms [17,18]. Outbreaks among cats in Korea, France and Poland all show E627K [20,59, 60]. Sporadic detections in wild mammals such as foxes, bears and otters also reveal combinations of some or all of E627K, D701N and T271A, which is a testament to the ease at which polymerase mutations can arise *de novo* from a spillover from wild birds [7,8, 14, 61, 62]. Following an initial spillover, polymerase adaptation combined with a high-density environment makes mass transmission possible. Once a single adaptive mutation arises, this may be enough for them to become dominant, explaining why there are a range of adaptive mutations observed in these outbreaks aside from E627K.

### Further polymerase adaptations

The extent to which other mutations contribute to influenza polymerase mammalian adaptation is not clear, particularly in a landscape of well-characterized mutations which support co-opting of ANP32 proteins. Recent studies have shown that changes in PA and PB1 can lead to mammalian adaptation. Several of these polymerase mutations occur in the symmetric dimer interface, reducing the presence of the symmetric dimer [45]. This potentially increases the asymmetric polymerase dimerization with ANP32, thus allowing for the co-opting of suboptimal ANP32 homologues such as ANP32E [63].

Changes at residue 86 of PA are seen in marine mammals (M86I) and in mink from Spain (M86T). Structurally, this site is in the PA-PB2 interface [64]. These are not well-characterized mutations, although *in vitro* experiments show that T86I and M86V boost polymerase activity in H5N8 and H5N1 viruses, respectively [65,66]. The mutations tested are not identical to the mammalian mutations observed but could be inducing similar conformational changes altering dimerization [45]. At the time of writing, M86I has been seen in a third spillover into cattle from wild birds without other clear mammalian adaptations [67].

The accessory proteins PA-X and PB2-F2 may also contribute to increased pathogenicity or virulence [68,69]. The PB1-F2 mutation N66S has been seen in bears, rats and South American marine mammals [24,25, 70], which increases the virulence of H5N1 in mice [71]. Generally, though there is limited evidence of mammalian adaptations emerging in these segments, this could be an area of interest for further research and surveillance.

Overall, mutations in the polymerase are driven by the need to adapt to host ANP32 proteins. Spillback of mammalian-adapted viruses back into birds was seen from cattle viruses with M631L into blackbirds, grackles and domestic poultry without any clear loss of fitness. Although these mutations are not fixed in the wild bird populations, lab experiments normally show no loss of fitness with mammalian mutations in avian cells. Perhaps the clearest example of this is in the Antarctic region and South America, where PB2 Q591R and D701N have been detected in multiple mammalian and avian samples. Complex ecological networks and transmission chains, including birds scavenging from dead mammals, can support the maintenance of mammalian-adapted viruses in wild bird populations [22,72]. Mammalian markers in avian samples, such as D701N, often signify that the virus has recently been in a mammal, but these mutations can occasionally arise naturally in birds, highlighting the importance of surveillance of mammalian markers in avian species.

### Haemagglutinin

The HA is found on the surface of the virion and binds glycans terminating in sialic acid, allowing for attachment and entry into the host cell [73]. Avian viruses preferentially bind *α*2,3-linked sialic acid and consequently cause a gastrointestinal disease in birds as this is the phenotype of receptors in the avian intestinal tract. In humans however, influenza primarily attacks the upper respiratory tract (URT) and lungs, where epithelial cells have largely *α*2,6-linked sialic acid, though *α*2,3-linked sialic acids are found deeper in the lungs. Other species including pigs, seals and ferrets contain a combination of sialic acid in their airways [74,75]. The distribution of different sialic acids has not been fully catalogued for most tissue types and species. The sialic acids present are ultimately a major determinant of influenza infection and transmission.

**Fig. 4.**
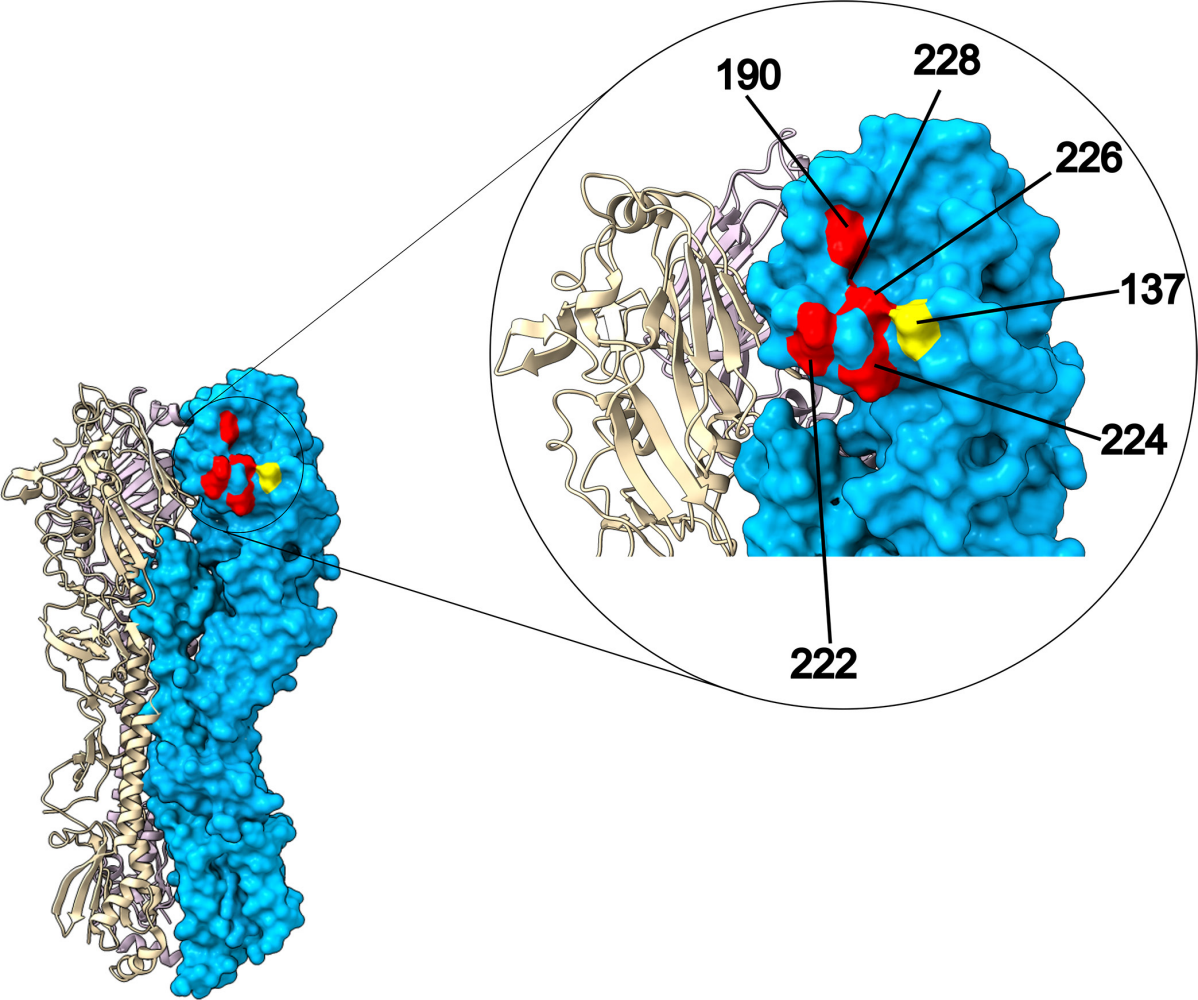
H5N1 A/Texas/37/2024 HA homotrimer. One HA monomer is highlighted in blue with key residues that confer *α*2,6 sialic acid preference highlighted in red. Bovine-specific site change is highlighted in yellow. PDB: 9DIP [43]. Protein structure graphic created using ChimeraX version 1.9 [149].

For AIVs to successfully evolve human-to-human transmission, they must switch their receptor preference from *α*2,3-linked to *α*2,6-linked sialic acid. The main mutations in the H5 HA that confer *α*2,6 preference have been experimentally identified as E190D [76], and in the 220 loop, Q226L, N224K and G228S [76,77] (*note: standard mature H3 numbering used throughout unless otherwise specified*). These residue sites are in the receptor binding pocket and conformationally improve binding affinity with *α*2,6-linked receptors (see Fig. 4). Mutations in the 220 loop are essential and molecularly accommodate a change in receptor through introducing hydrophobic changes and different side chains. Changes at residue 190 are epistatic and increase binding affinity by shortening the 190 side chain whilst remaining acidic, reducing interference with the *α*2,6-linked receptor [77]. Mutations at 190 and in the 220 loop also facilitate airborne transmission in ferrets, both together or individually to a lesser extent [78,80]. Q226L is the most important of these mutations, with a recent study also confirming that Q226L can uniquely switch receptor binding preference from *α*2,3 to *α*2,6 when using the first bovine-associated human H5N1 isolate as a backbone [81].

High-throughput methods such as deep mutational scanning are an increasingly important tool in identifying adaptive mutations before they emerge in nature and contribute to our ability to risk assess viruses in real time. Dadonaite *et al*. assessed the impact individual mutations have on receptor binding and confirmed key residues where mutations promote *α*2,6 sialic acid binding of H5 HA, namely, 137, 190, 193, 224, 225, 226 and 228 [80].

As well as changing receptor preference, to adapt to humans, AIVs must also become more pH and temperature stable to successfully persist in different environments and infect respiratory airways. During cellular entry, once the virion has been taken into the cell by endocytosis, cleavage of the HA at low pH further fuses the viral envelope to the cell endosomal membrane, resulting in the release of the viral genome into the cell [81]. The pH within a respiratory droplet can be low, and, for effective respiratory transmission, increased HA stability at low pH is needed to prevent irreversibly cleaving too early [81]. Mutations are therefore required to increase stability so that virions can persist in the environment and the URT of humans longer [1]. Deep mutational scanning confirmed previously known stabilizing mutations such as Y17H, H24Q and T318I [78,79, 82]. Novel sites where changes greatly increase the mean stability were also identified through this method and include 62, 82a, 109, 262 and 334 [79]. Stabilizing mutations are generally found in the stalk domain of the HA, rather than the receptor binding domain [75,83]. However, the precise molecular mechanism behind the above stabilizing mutations remains unresolved.

Mutations of concern which favour *α*2,6-linked receptor binding have not been seen in the B3.13 outbreak in cattle or associated human cases. Reassuringly, binding assays of early HA mutations seen in cattle showed no increased binding to human-like *α*2,6 sialic acid [82]. Additionally, the same work shows that HA activation with these mutations is approximately pH 5.9, which is above the mammalian-like activation pH of 5.5, suggesting an avian-like HA phenotype [82]. These mutations also either reduced thermostability or had little impact compared with the WT, confirming at this point that the virus is not yet stable at lower pH [82]. Physiologically, there is a high prevalence of *α*2,3-linked receptors in bovine mammary glands where infection seems to be concentrated, asserting a low selective pressure to adapt to binding human-like *a*2,6 sialic acid. Bovine trachea cells are also predominantly *α*2,3-linked receptors [75], with recent work also showing high levels of NeuGc-based receptors, further reducing the selection pressure to human-like NeuAc *α*2,6 sialic acid [83].

In contrast to other published results, Eisfeld *et al*. found that bovine B3.13 bound both avian *α*2,3 and human-like *α*2,6 sialic acid, and they showed limited airborne transmission between infected ferrets [84]. Case report analysis of test-positive cases of H5N1 in the USA has shown some low-level detections of viral RNA in nasopharyngeal swabs [85], but most infections have presented with conjunctivitis explained by a high proportion of *α*2,3 sialic acid being present in the eye [86]. However, given that we have not seen human-to-human transmission and cases have presented with ocular rather than respiratory symptoms, it is likely that the cattle strain does not yet bind *α*2,6 sialic acid effectively. Similarly, the H7N7 outbreak in poultry in the Netherlands in 2003 resulted in 89 associated human cases, of which most presented with conjunctivitis with only 1 fatality [87]. Interestingly, polymorphisms at 226Q/H and 190E/D were seen in the severe human case in British Columbia [31], Canada, as well as the fatal human case in LA [32], USA – both of the distinct D1.1 genotype. This could suggest that current H5s do not bind *α*2,6 sialic acid, but in sustained respiratory infections, there is selective pressure to bind *α*2,6 sialic acid. There does not seem to be a stabilizing effect from these mutations [80], nor at other key receptor binding sites 222 and 224, which may imply that additional mutations will be required to support droplet transmission. Why these changes have occurred in cases of D1.1 vs B3.13 is unclear despite many more human cases of B3.13, though we speculate it could be linked to the transmission route or severity of infection.

In addition to the HA mutations listed above, a suite of other mutations has been observed in the HA across other mammalian outbreaks (e.g. S128P, S137A, S158N, T160A, T192I and V214I [88,92]). Some of these are known to increase *a*2,6 sialic acid binding affinity and from a human health perspective have been flagged as important for risk assessing novel AIVs [88]. T160A and S158N are characteristic of clade 2.3.4.4 avian viruses but have not necessarily arisen *de novo* in mammals [93]. T160A and S158N are found in marine mammals [24,25], cats [94] and also dairy cattle along with S137A, which was also found in foxes [11,95]. Both T160A and S137A improve binding to both *α*2,6 and *α*2,3 sialic acids [93,96]. Structurally, 137 is close to the key mutations in the 220 loop (see Fig. 4) and has also been highlighted through deep mutational scanning to increase *a*2,6 sialic acid binding. S137A and T160A were observed in human 2.3.4.4b H5N6 isolates, highlighting that they can cause severe disease even in the absence of Q226L [97,98]. However, the extent to which these mutations promote mammalian spillover or support URT infections in humans remains unclear.

Ultimately, whether the HA needs to evolve to productively infect mammals depends on species-specific receptor types and distributions. Mammal-to-mammal transmission can occur in many environments, despite no major HA adaptations. Spread in dairy cattle was with an HA that preferentially binds *α*2,3 sialic acid, and this was also the case in mink, cats, seals and sea lions, though we do not fully know the receptor profiles in these species. For human-to-human transmission, preference for *α*2,6 must evolve, and spillover into swine or other species that have mixed sialic acids would present an opportunity for this receptor preference switch.

It is possible that epistatic interactions are needed to support both *α*2,6 binding and the increased stability necessary for a human transmissible virus. These changes have yet to be seen together in the current H5 background. There may be a trade-off between binding and stability, which has prevented the emergence of such changes accumulating simultaneously [99], presenting an evolutionary fitness valley for the virus to overcome to become transmissible in humans. However, the concern remains that single nt point mutations at key sites can swap the HA preference, which along with stabilizing mutations manifests the alarming reality of how close this virus is to preferentially binding human-like receptors and becoming capable of transmission.

### Further mammalian adaptations

#### Neuraminidase

The neuraminidase (NA) is also a surface protein and cleaves sialic acid on the surface of the infected cell, allowing newly formed virions to be released from the cell [100]. NA also cleaves ‘decoy’ sialic acid receptors found in soluble mucus, ensuring virions do not become trapped.

The NA stalk length is proposed as a key characteristic that may contribute to increased transmission [101]. A long stalk NA is proposed to overcome mucus inhibition, reaching through to the sialic acid receptors on the cell surface more effectively in humans [102]. Using NA from a human H1N1, short and long stalk NA H5N1 viruses were compared in human epithelial cells and in a ferret model. Virus replication and transmission were both reduced in the short stalk model. Conversely, in mice and chickens, a short stalk has been shown to be more advantageous to the virus [103], indicating that host-specific nuances to its structure are important in its fitness. GsGd H5N1 viruses originally had a long stalk NA; however, this was lost following its emergence and circulation in domestic poultry [101]. Contemporary 2.3.4.4b H5N1 viruses have a long NA stalk with recent analysis suggesting that the glycosylation characteristics of the 2.3.4.4b HA have driven preferential pairing with long stalk NAs [104], which could also explain why this clade has been so successful in mammalian hosts [101,103].

The second sialic acid-binding site (2SBS) is an additional site on the NA, which can bind to sialic acid and is highly conserved among viruses of wild birds. There is limited consensus on the mechanism of the 2SBS. It is thought that it enhances NA activity by binding *α*2,3 linked receptors, bringing them closer to the main NA catalytic cleavage site [105]. The 2SBS may also directly improve HA binding with *α*2,3 sialic acids [106,107]. The 2SBS is lost in human pandemic lineages, however, indicating that it is redundant for human fitness given the shift of preference to *α*2,6 binding and may be detrimental in binding *α*2,6 sialic acids [105]. In N1 NAs, the key regions of the 2SBS are the 370, 400 and 430 loops [105]. Mutations S369I and I396M/V, which disrupt the 2SBS and make it more human-like, have been detected in isolates from the Spanish (I396M) [17] and Finnish [108] (I396V) mink outbreaks as well as the precursor European gull genotype, which has circulated since 2022 and spread through seabird colonies. However, it remains unknown why changes in the 2SBS emerged in birds unrelated to mammalian infections [17,109, 110]. Within cattle, some mutations close to these regions have also emerged (e.g. N366S, G382E, A395E and S434N) [27], but there is limited data on whether these mutations affect the 2SBS. It is important that NA and HA activity are balanced, and there is evidence suggesting that changes in either glycoprotein can drive mutations to improve the balance of HA-NA binding [105]. However, more research is needed on this to understand the evolutionary drivers of epistatic HA-NA mutations in this context [92].

Although there may be other changes in NA, there is limited data to inform on their function and whether they are adaptive. A combination of stalk length, 2SBS and parallel changes to HA which complement *α*2,6 binding may all play a role in NA adaptation.

#### Nucleoprotein

The major role of nucleoprotein (NP) is structural, encapsidating the genomic vRNA along with a viral polymerase forming the vRNP [111,112]. Host restriction factors MxA and BTN3A3 target the NP, and avian NPs require mutations to avoid restriction in human cells. MxA is a group of IFN-stimulated proteins thought to block vRNPs entering the nucleus and therefore replication. Mammalian adaptive NP mutations V100I, L283P and F313Y [113,114] have been shown to overcome this, though in doing so, there is a degree of attenuation as the nuclear import mechanism which MxA targets is both evaded and disrupted [113]. Further mutations Y289H and E53D have also been shown to boost MxA resistance [115], with G16D stabilizing NP, compensating for the destabilizing effect of the other mutations [113].

BTN3A3 is an IFN-stimulated gene and has been linked to restricting viral replication [116]. Human-adapted influenza strains are not sensitive to BTN3A3, whilst most avian strains are. NP mutations F313Y/V and Y52N/H/Q were identified to overcome BTN3A3 restriction, and F313Y can also overcome MxA restriction in combination with mutations listed above [116]. Structurally, sites 283, 313, 53 and 100 all sit close to each other on the surface of the NP head domain [116]. Changes at residue 52 are also involved in both MxA and BTN3A3 restriction escape [116].

In dairy cattle in the US [27] and European and North American pinnipeds, Y52H has been reported [117], whilst Y289F was identified in South American marine mammals [58]. Unlike key polymerase mutations, changes in NP have been less frequent and have not necessarily arisen *de novo*. For example, there are avian viruses with NP Y52H, which may be precursors to pinniped detections, and in dairy cattle, this change was also present in closely related avian sequences. A Y52N variant was seen in the outbreak in mink in Spain but was also present in the wild bird reservoir [17], suggesting a pre-adapted strain as a precursor to the mink outbreak. In dogs, horses and pigs, equivalent BTN3 homologues such as BTN1 do not inhibit AIV activity [116]. Despite this, changes in the same region of NP are seen across these species. This suggests that multiple host factors such as MxA and BTN3A3 target the same region of NP; thus, the same mutations are important in overcoming restriction [116]. Further work to determine host restriction across mammalian species would improve our understanding of adaptations to overcome generic and species-specific host restriction factors.

It is also possible that species-specific adaptations in NP emerge following extended circulation in humans. A good example is Q357K, a known signature of mammalian adaptation identified through bioinformatics as a marker of more than one human and swine pandemic lineage [118]. Experimental work in mice demonstrated that it significantly increases virulence, and polymerase activity data also shows that it increases polymerase activity in human cells [119]. The 357 residue is in the binding pocket of NP with RNA, explaining how it could impact replication. However, it has not been seen as an adaptation during mammalian H5N1 outbreaks or human cases despite its prevalence in human seasonal and pandemic viruses. Many potentially pre-pandemic strains have pre-adapted NPs, avoiding host restriction, but it is unclear to what extent these mutations are being selected on in birds.

#### Segments 7 and 8

Both segment 7 and 8 mRNAs are spliced by host factors to encode two different viral proteins per segment [120,121]. Segment 7 encodes the structural matrix proteins M1 and M2. M1 is very abundant, forming a layer beneath the viral lipid bilayer, and is associated with viral assembly and export of vRNP [122]. M2 is an ion channel proton pump that spans the envelope, controlling the pH and stabilizing the virus at various stages of replication [123,124]. Studies of H5N1 in mice have shown that the two mutations N30D and T215A in M1 have a polygenic effect and contribute to increased lethality [125,126]. More specifically, these changes interfere with SUMO1 (a post-translational modifier) interactions, preventing SUMOylation of the M1 protein, which is key for its stability and ability to associate with vRNPs [122,126]. N30D and T215A are seen quite commonly in the mammalian outbreaks: bears and carnivores in France [13], foxes in the USA [11] and porpoise in Sweden (Y215A) [16]. However, these mutations existed in the circulating 2.3.4.4b viruses and were not a product of *de novo* adaptation. The sea lions in Brazil also had the I43M mutation (also in the avian reservoir) in addition to N30D and T215A, which has been experimentally shown to cause higher virulence in avian and mouse models. Although the mechanism of this adaptation remains unknown, it has been hypothesized that the increase in pathogenicity is linked to thermal stability and virion shape [127].

Segment 8 of AIV also codes for two separate proteins: NS1 and NEP (also known as NS2). NS1 antagonizes the host response, primarily by interfering with the IFN response by binding RNA and cellular signals [128]. NEP is involved with nuclear export of the vRNA along with M1 [129]. There are few changes in NS1 or NEP that have arisen specifically in mammals during recent outbreaks. Some mutations in NS1 associated with mammalian adaptation, e.g. P42S [130], L103F and I106M [131], are found in avian 2.3.4.4b viruses. 103F and I106M are thought to interact with antiviral factors triggering IFN production by binding host cleavage and polyadenylation specificity factor, preventing restriction-associated mRNA export out of the nucleus [131,133]. Experimentally, introducing these human mutations to a 1997 H5N1 NS1 significantly increased virus replication [133,134], but as contemporary H5N1 viruses commonly carry these mutations, these mutations are not indicative of mammalian infection. In addition, mutations in NEP have been shown to allow avian viruses to overcome host restriction caused by ANP32 [135]. NEP promotes vRNP formation and the interaction of vRNPs with human ANP32s independently of its ability to export vRNAs [135]. This has been shown to be dependent on a SUMO-interacting motif in NEP [135]. In NEP, mammalian adaptive mutations M16I, Y41C and E75G in particular are associated with increased polymerase activity but have not been observed in outbreaks [136].

NEP is increasingly thought to be an important determinant of tropism [136], by mediating the shift in viral transcription to replication [136,138]. Changes in splicing activity of segment 8 will impact the ratio of NS1:NEP, and the accumulation of NEP will affect the ability to use human ANP32. In human hosts, avian viruses produce less NEP compared with NS1, which could be an important aspect of host adaptation [120]. Higher levels of NS1 may also lead to increased pathogenicity through strong inhibition of host IFNs by NS1 [120,121]. Splicing of segment 7 in avian viruses is also dysregulated in mammalian cells, resulting in overexpression of M2 proton channels. This reduces viral replication as essential host cellular functions involving lysosomal fusion are inhibited [139]. Human-adapted viruses do not have this problem however [139]. It is unknown the extent to which segment 7/8 splicing is contributing to spillover into mammalian species. Mutations in the polymerase are more common and more effective than NEP at overcoming host restriction due to ANP32. However, variation in NEP may be responsible for the initial replication of some avian viruses in mammalian cells in the absence of polymerase mutations.

### Neurotropism and pathogenicity

2.3.4.4b infections have shown a marked neurotropism in mammals with cats, zoo tigers and foxes severely affected [140]. In 2024, goats in the USA were found to be infected with the same clade of H5N1 and displayed neurological symptoms [141]. Whether there is underlying biology that makes these species particularly susceptible to neurotropic symptoms or how influenza viruses differ in their neurotropic potential remains unknown. The HA mutation T132A seen in cats is associated with immune escape [142], but it is not thought that this change or receptor distribution is the reason cats display neurotropism as both types of *α*2,6 and *α*2,3 sialic acid receptors were found in abundance in the brain. This does raise the question of whether cats may therefore be good ‘mixing vessels’ for adaptation to human-like receptors, particularly as they are companion animals often in close contact with people [37].

Another important consideration is how the mode of transmission affects severity. Infecting non-human primates with cattle B3.13-like viruses via different routes showed that the orogastric route did not result in as severe disease as respiratory routes [143]. Given the risk of contaminated dairy products, infection severity mediated by the mode of transmission could be a factor in the extent humans are infected. In cats and other mammals however, the orogastric route may lead to more severe infection and would be an interesting area of further research in the context of species and tissue-specific glycan receptors. Whilst many individual mutations have been associated with increased pathogenicity (often due to increased viral replication [144,145]), the biochemical mechanisms of differences in pathogenicity between influenza viruses remain underexplored [146,147].

At the time of writing, D1.1 has recently been detected emerging in dairy herds for the first time [28,57]. Anecdotal evidence of two severe cases may suggest that D1.1 could be more severe than B3.13; however, there is limited evidence to support this given many unknowns about comorbidities and transmission routes. Detailed and broad investigation is needed to tease apart how mode of transmission, underlying virus, host biology and case ascertainment may influence our understanding of virus severity.

### Informed guesswork or a roll of the dice?

With our understanding of how H5N1 viruses adapt to mammalian and human hosts, are we now able to reasonably speculate on the risk of H5N1 further adapting to become a human pandemic strain?

Recapping the ‘pandemic staircase’ introduced earlier (Fig. 2), following a mammalian infection, the first step is one or more polymerase mutations adapting to ANP32. The next step, to achieve human-human transmission, requires mutations inducing HA changes to switch receptor binding and increase environmental stability. The final step is adaptation towards host evasive mechanisms and further fine-tuning [43]. Current evidence suggests that the 2.3.4.4b clade has and can easily overcome the first step as mutations arise readily in the polymerase almost instantly following spillover, as demonstrated by the frequent occurrence of key mutations such as E627K, D701N, Q591K and M631L in PB2 across multiple species and settings. We know that only minor changes in the polymerase were needed for it to spread within populations of cattle, mink and marine mammals.

Changes to the HA remain more speculative however, with a stable and transmissible virus yet to be seen in humans. Putative changes affecting receptor binding have been seen including Q226H and E190D in recent human cases, although these mutations have not been fixed within a host [31,32]. Further combinations of mutations promoting both stability and human receptor binding may emerge, allowing the virus to fulfil the second criterion. Species-specific receptor differences present key species barrier but also potentially stepping stones to a human-adapted HA.

Compared with the polymerase and viral surface proteins, internal genes NP, NS and M pose less of a serious initial threat in driving pandemic potential. Specific changes in these genes are important in fine-tuning fitness and evasion of host restriction – the last ‘step’ in the evolution of a pandemic strain. However, many avian strains naturally have humanizing mutations allowing them to escape potential restriction and may be more capable of initially infecting humans. Should further sustained transmission evolve, we may see viruses already primed for host evasion expediting the virus’s ability to gain traction in the human population. Another shortcut to mammalian adaptive mutations is the reassortment of genes with other circulating AIVs. By swapping segments, the contemporary H5N1 viruses could acquire many of the mutations needed in the polymerase, internal segments and to some extent the NA for a human-adapted virus. Since 1918, all influenza pandemics have been products of reassortment events [148].

Our understanding of the mechanism behind adaptation allows us to predict whether a virus is adapting to human hosts and assess the risk it poses to public health. Deep mutational scanning coupled to the potential of AI models promises a future where we may have greater predictive power over which mutations and viruses pose the greatest threat. Further research should focus on key unknowns such as the determinants of severity across species and the importance of modes of transmission.

The prevalence of infection across so many species creates a perfect storm for exposure to new hosts and the potential for further adaptive mutations to emerge. There is also a significant risk of reassortment with other circulating influenza viruses, which may fast-track the acquisition of human adaptive mutations from seasonal lineages or other AIVs. Ultimately, time will tell if this virus evolves into a pandemic virus. However, we can be certain that limiting transmission and the opportunities avian viruses have to adapt is important to prevent existing and new mammalian epidemics.

## Supplementary material

10.1099/jgv.0.002109 Table S1.

## Abbreviations

AIV: avian influenza virus
CFR: case fatality rate
cRNA: complementary RNA
HA: haemagglutinin
HPAI: high pathogenicity avian influenza
LCAR: low complexity acidic region
LPAI: low pathogenicity avian influenza
NA: neuraminidase
NP: nucleoprotein
2SBS: second sialic acid-binding site
URT: upper respiratory tract
vRNA: viral RNA
vRNP: viral ribonucleoprotein
