# Supplementary material for: H5N1 2.3.4.4b: a review of mammalian adaptations and risk of pandemic emergence
Source: J Gen Virol. 2025 Jun 4;106(6):002109. doi: 10.1099/jgv.0.002109 (PMC12137919; doi:10.1099/jgv.0.002109)
Supplement: Table S1. [file jgv-106-02109-s001.pdf]

Supplementary materials to the manuscript: H5N1 2.3.4.4b – a review of mammalian adaptations and the risk of pandemic emergence

Table S1. Species and silhouette key for figures in Figures 1 & 2. Silhouettes obtained from PhyloPic (www.phylopic.org).

Silhoutte	Species
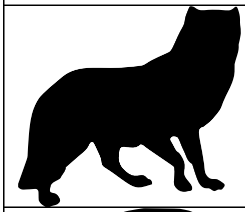	Arctic Fox
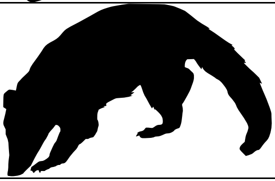	Coati
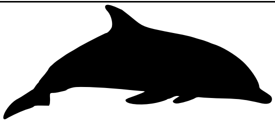	Dolphin/Porpoise
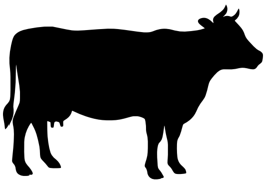	Dairy Cattle
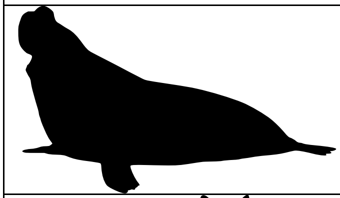	Elephant Seal
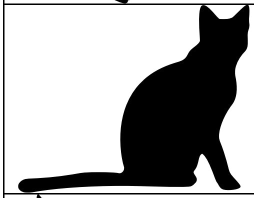	Cat
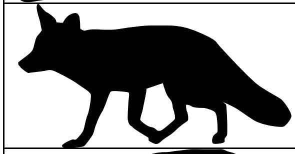	Fox
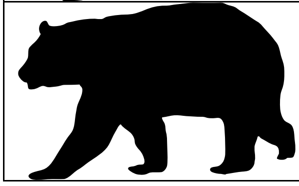	Bear

Silhoutte	Species
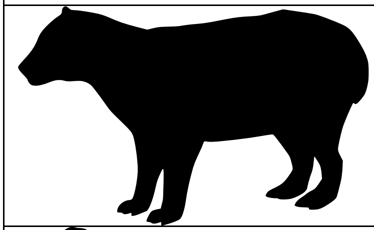	Bush Dog
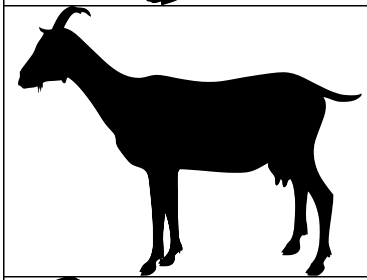	Goat
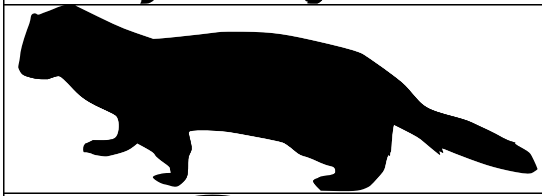	Mink
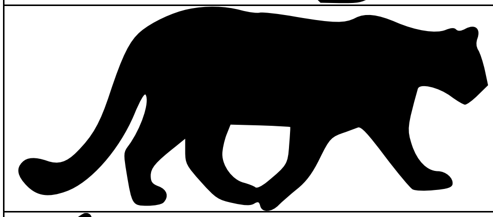	Puma
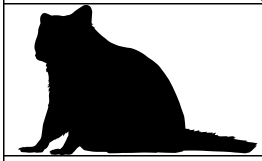	Racoon
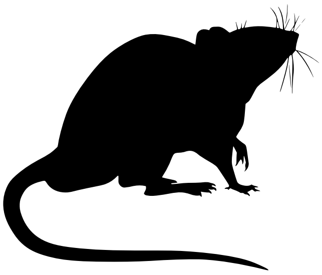	Rat
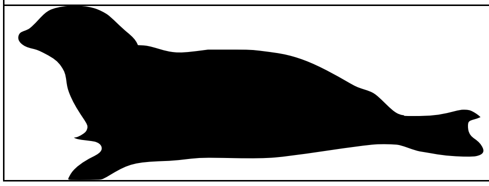	Seal

Silhoutte	Species
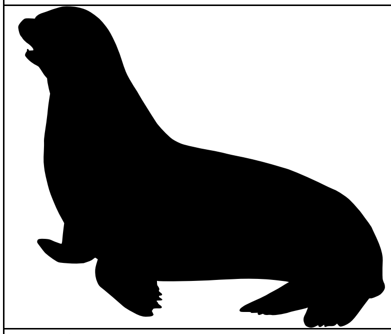	Sea Lion
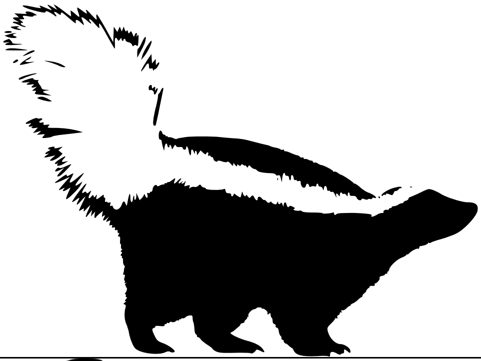	Skunk
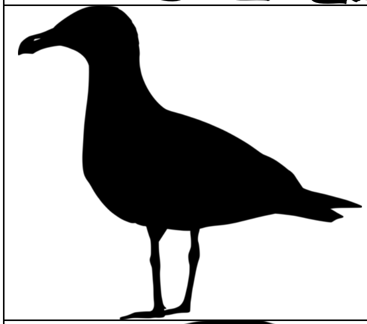	Gull
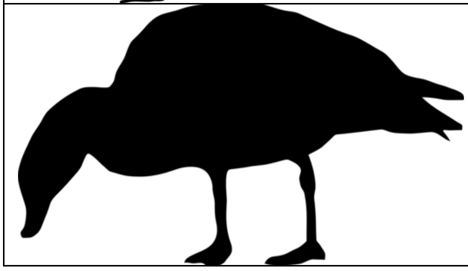	Duck
